# Cross-border spatial accessibility of health care in the North-East Department of Haiti

**DOI:** 10.1186/s12942-018-0156-6

**Published:** 2018-10-25

**Authors:** Dominique Mathon, Philippe Apparicio, Ugo Lachapelle

**Affiliations:** 1 Environmental Equity Laboratory, INRS Centre Urbanisation Culture Société, 385, rue Sherbrooke Est, Montréal, Québec H2X 1E3 Canada; 2 Département d’études urbaines et touristiques, Université du Québec à Montréal, Case postale 8888, Succursale Centre-Ville, Montréal, Québec H3C 3P8 Canada

**Keywords:** Spatial accessibility, Health care, Enhanced two-step floating catchment area, Border, Haiti, Dominican Republic

## Abstract

**Background:**

The geographical accessibility of health services is an important issue especially in developing countries and even more for those sharing a border as for Haiti and the Dominican Republic. During the last 2 decades, numerous studies have explored the potential spatial access to health services within a whole country or metropolitan area. However, the impacts of the border on the access to health resources between two countries have been less explored. The aim of this paper is to measure the impact of the border on the accessibility to health services for Haitian people living close to the Haitian-Dominican border.

**Methods:**

To do this, the widely employed enhanced two-step floating catchment area (E2SFCA) method is applied. Four scenarios simulate different levels of openness of the border. Statistical analysis are conducted to assess the differences and variation in the E2SFCA results. A linear regression model is also used to predict the accessibility to health care services according to the mentioned scenarios.

**Results:**

The results show that the health professional-to-population accessibility ratio is higher for the Haitian side when the border is open than when it is closed, suggesting an important border impact on Haitians’ access to health care resources. On the other hand, when the border is closed, the potential accessibility for health services is higher for the Dominicans.

**Conclusion:**

The openness of the border has a great impact on the spatial accessibility to health care for the population living next to the border and those living nearby a road network in good conditions. Those findings therefore point to the need for effective and efficient trans-border cooperation between health authorities and health facilities. Future research is necessary to explore the determinants of cross-border health care and offers an insight on the spatial revealed access which could lead to a better understanding of the patients’ behavior.

## Background

The geographical accessibility of health services is an important issue in public health and for improved health outcomes, especially in developing countries [1–6]. During the last two decades, numerous studies have explored the potential spatial access to health services within a whole country or metropolitan area [7–10]. Scholars have also analyzed cross-border mobility for health care in several diverse contexts [11–24]. But fewer studies address the impact of an international border and its openness on the spatial access to health care resources [25, 26].

The concept of borders has been evolving throughout the years from their being seen as barriers to their being considered as contact zones, but regional integration and border openness have been questioned in several contexts [27–32]. Studies analyzing cross-border mobility for the use of health care services emphasize the uniqueness of the different border contexts and the importance of the direction of flows [16, 24]. Cross-border mobility for health care access may be explained by a variety of factors. It depends on the various individuals’ situations and needs. It may be motivated by dissatisfaction with health care provision in the home country or by actual deficiencies there. A lack of coverage (in terms of health care insurance) or a quest for specialized health care may influence individual choice. Glinos et al. [12] indicate that, during the decision-making process, patients balance factors such as proximity, family support and social ties. Affordability, availability and quality of care are also determinants. Analyzing patient mobility between Laos and Thailand, Bochaton [14, 15] demonstrates the importance of well-established mobility practices as well as social networks among border populations in the seeking of cross-border health care. Social networks are also considered by Dione [16] as one of the determinants of patients’ cross-border mobility in four African countries sharing a border. Proximity (physical accessibility) is also one of the main determinants of patient mobility in the very different contexts of European [11] and African countries [16].

Access to health care is multidimensional, and most of the studies on patients’ cross-border mobility for health care access have used the seminal framework developed by Penchansky and Thomas [33]. These authors consider five dimensions in order to measure “the degree of fit between the clients and the system” [33]. Two of these dimensions are spatial: (1) availability (adequacy between the supply and the demand); and (2) accessibility, or the location of the supply relative to the location of the clients. The other three are aspatial and reflect socioeconomic and cultural factors: (1) accommodation, or the adequate matching of the supply organization with the clients’ abilities and perceptions; (2) affordability, or the prices of the services relative to the clients’ income or ability to pay; and (3) acceptability (clients’ and providers’ attitudes toward one another). These dimensions may act as either facilitators or barriers.

Regarding spatial accessibility, scholars define this in terms of the possible use of the services (potential accessibility) and their actual use (realized accessibility) [34, 35]. This differentiation between potential access and realized access makes it possible to better identify the barriers to or facilitators of access. The extent of the spatial separation between supply and demand can therefore be analyzed. In this article, we focus on potential spatial accessibility in a borderland context. The border acts either as a geographical constraint or as a facilitator.

Our hypothesis is that accessibility varies depending on the level of border openness. In addition, the lack of services (push factor) in Haiti and the more attractive supply (pull factor) in the Dominican Republic may lead to polarized flows in a push/pull dynamic.

The aim of this paper is to evaluate the spatial accessibility of health care services for Haitians living along the Haitian-Dominican border, and to measure the impact of this border on their health care access using the well-known E2SFCA method.

### The Haitian-Dominican border

The Haitian-Dominican border inherited from the colonial period has given rise to a “double insularity” [36, 37] that has been settled through a long process of social and spatial differentiation as well as ideological distancing [36–38]. Both countries have forged and asserted their particular national identities through their respective histories and struggles to achieve the construction of their own nation state [37, 39]. The discontinuities (territorial, cultural, socioeconomic and political) are therefore quite visible at the Haitian-Dominican border [40, 41]. An entire apparatus (gates, military control on the Dominican side, etc.) is in place to mark and create this distance [41–43]. At the same time, the relative and recent border opening has given rise to a transitioning process which is redefining the function of the border as moving toward a “space of coexistence and cooperation” while sustaining asymmetrical and conflicting interactions along the border line [40, 42, 44, 45].

Officially (since 1987), the border has been opened during the day and closed at night. There are four official entry points and several informal crossing points, the number of which is not precisely known [38]. These informal crossing points underscore the permeability of the border as well as the complexity of the cross-border mobility [43]. The flow of the population may be constrained by different conflictual situations: a national decision (epidemiological surveillance, control of smuggling, etc.) or a particular local situation (protest about Dominican soldiers’ aggressive behaviours, protest over national decisions, protests from Haitian or Dominican traders, etc.) [44]. From 2000 to 2016, the border was closed a number of times for varying numbers of hours or days. But the intensity and importance of the commercial exchanges for both countries, at different levels, may act as a leverage for conflict settlement.

Cross-border movements from both sides have existed since the colonial period, but Haitian labour flows started in the early twentieth century with the North American occupation of both countries [38, 43, 46–48]. Various mechanisms are in place in the Dominican Republic to regulate such flows (illegality of the Haitian work force, massive deportations, etc.) [38, 48]. According to the recent survey on migration, more than 80% of immigrants in the Dominican Republic are Haitian [49, 50]. The importance of Haitian labour for the construction industry as well as for the agricultural sector is well documented [38, 43, 46, 47, 49–51]. Some studies [38, 46] have revealed a “feminization of Haitian migration flows.” Others [42] have emphasized the difficulty of distinguishing between irregular migration, smuggling and trafficking.

On the other hand, there is some evidence that the percentage of Haitian immigrants using health care facilities is higher than for other immigrants [49]. Furthermore, during the last two decades, the Dominican Republic has been used to channel international aid to Haiti. Montiel et al. [46] emphasize the differential impact of this, including, for example, the reinforcement of the Dominican health care system at the expense of the Haitian one. They consider this to be a factor that could have encouraged a growing number of Haitians to cross the border in search of health care [46]. Their comments are in line with evidence from other studies addressing cross-border health care mobility in different contexts [12, 15, 16]. But beyond the significant and quite systematic health outcome disparities between both countries (Table 1), what are the differences between the two health care systems?

**Table 1:** Basic health indicators for Haiti and the Dominican Republic

Health indicators	Haiti	Dominican Republic
Life expectancy at birth (2016) *	63.3	73.9
Men	61.2	70.8
Women	65.5	77.1
Mortality rate of the under 5 years (probability of death before age of 5 per 1000 live births, 2016)**	67	30.7
Maternal mortality ratio (per 100,000 live births, 2014)***	359	92
New HIV infections among adults 15–49 years old (per 1000 uninfected population, 2015)****	0.21	0.36
Births attended by trained personnel (%)	50.0^a^	68.6^b^
Skill health professionals density (per 10,000 habitants)	6.5^c^	28.2^d^

*Sources*: *World Development Indicators. World Bank Group at databank.worldbank.org

**Estimates Developed by the UN Inter-agency Group for Child Estimation (UNICEF, WHO, World Bank, UN DESA Population Division) at childmortality.org

***WHO, UNICEF, UNFPA, World Bank Group, and the United Nations Population Division. Trends in Maternal Mortality: 1990–2015. Geneva, World Health Organization, 2015

****2015, *Source*: UNAIDS/WHO; estimates 2016

^a^2015

^b^2014; PAHO/WHO, Health in the Americas—Summary: Regional Outlook and Country Profiles, 2017

^c^*Source*: MSPP, 2012

^d^*Source*: 2005–2013, WHO Global Health Workforce Statistics database

### Main characteristics of the public health care systems in Haiti and the Dominican Republic

The health care system in most Latin American and Caribbean countries is segmented, with a variety of financing structures and affiliation types. It is also fragmented, with a supply offered by many institutions (public and private) and facilities that are not well integrated into the health care network [52]. This fragmentation and segmentation exacerbate inequities in access [52], which is also the case in Haiti [53] and the Dominican Republic [53, 54].

#### Reforms of the health care system: Access to health care and equity

During the last two decades, both countries—like most Latin American [53, 54] and Caribbean countries [55]—have been involved in an ongoing process of reforming their health care sector. These reforms are intended to improve health outcomes and to reduce health inequities. They are based on the following principles: a regulatory role for public health institutions, multisectoral production of health care, universal access, equity and solidarity, and efficiency and efficacy of the health care system [56–59]. Changes have been made in the structure and organization of the public health care system in both countries in order to improve access to health care and especially to primary care. Nevertheless, the pace and the implementation of such reforms have fluctuated from one side of the border to the other [55].

In the Dominican Republic, the reform has been the starting point for universal access to health care [54]. Catchment areas have been defined to maximize resource allocation for primary care as well as for equity. Citizens must be assigned to or registered in a Primary Care Unit (*Unidad de Atención Primaria*). But the coverage is still deficient (less than 50% of the population was covered in 2012), with disparities found among different socioeconomic groups (the poorest have limited access to health care) and also between rural and urban areas [54].

In Haiti, changes have also been made to improve coherence with administrative boundaries and respect for the equity and universality principles included in the health reform [60]. But the Haitian health system still faces complex organizational and institutional challenges [55]. Moreover, data from the *Enquête Mortalité, Morbidité et Utilisation des Services* (EMMUSV) highlight the lack of coverage: less than 5% of the respondents [61] have health care insurance. As for the Dominican Republic, wealthier and urban people have more access, which means that any form of equity is still largely incomplete [61, 62].

#### Organization of the health care system

**Fig. 1:**
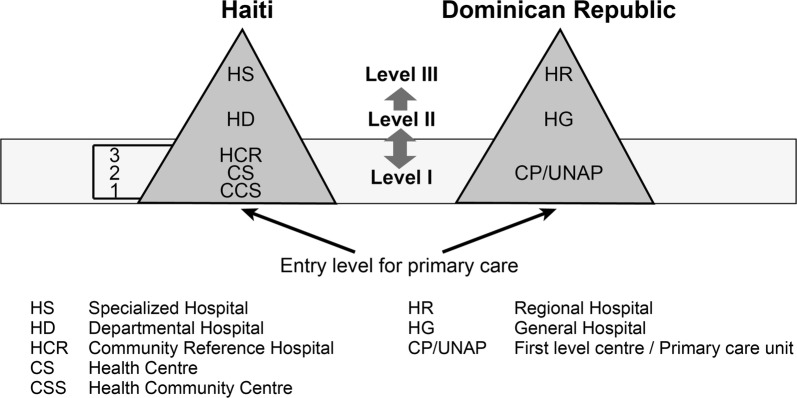
The three-tiered health care system of Haiti and the Dominican Republic

Both countries have a three-tiered health care system [56, 57, 60, 63, 64], but with some specific differences, as shown in Fig. 1. The pyramidal model is organized according to three levels of complexity: primary, secondary and tertiary. It is designed to break away from the existing hospital-centred structure in order to improve the population’s access to primary care. The reference and counter-reference system allows patients to transit within the system from the entry point to specialized services when required.

The primary level consists of outpatient services and community care. The first level therefore offers basic health care (minimum service package) and prevention and promotion activities. One of the main organizational differences between the Haitian and Dominican health care systems is found at this level. In Haiti, the primary level is subdivided into three parts. It includes different kinds of facilities located in distinct territorial entities: (1) Health community centre located in the *Section communale* (the smallest territorial division) and offering ambulatory care and prevention and promotion activities; (2) Health centre in the *Commune* delivering preventive and curative care, including normal childbirth; and (3) Community Reference Hospital (HCR) in the *Arrondissement* providing a range of care including sensitive interventions requiring specialists in internal medicine, surgery, pediatrics, obstetrics and gynecology. However, the official documents are somewhat confusing, as two of them [57, 65] consider only two subdivisions and others [60] mention three. Either way, the subdivision appears to be the Haitian health authorities’ response in order to accommodate the prevailing in terms of primary care facilities and to carry out the transition process toward the mainstream pyramidal model [60, 65].

It is important to emphasize that, in the Dominican Republic, each citizen is assigned a Primary Care Unit near their home (*Unidad de Atención Primaria*—*UNAP*) regardless of their insurance system [59], which is not the case in Haiti. Moreover, these units offer the same range of services as the first two Haitian first-level subdivisions.

The facilities of the second level (General Hospital in the Dominican Republic, whether administered at the municipal or provincial level, and Departmental Hospital in Haiti) offer basic specialized care in both countries. The services offered by the third level cover all contingencies during hospitalization and attend to the most complex cases.

#### Binational cooperation in health

The Haitian health master plan (2012–2022) considers reinforcing coordination with the Dominican Republic in order to reduce health issues in the epidemiological field in the borderland regions. It also seeks to develop relevant strategies and partnerships in the management of infectious diseases. There is a binational agreement for the control of tuberculosis aimed at successful coordination of the actions undertaken in the borderland regions and mainly targeting migrants, the populations of the *bateyes* (settlements around sugar mills where Haitian migrant workers live in very precarious conditions) and of the industrial areas, as well as those living in the borderland regions. In the case of natural disasters (floods in 2004 and the earthquake in 2010), the Dominican health facilities have supported the Haitian population by offering medical services to those needing them [44, 66, 67]. There are also coordinated vaccination campaigns in the borderland regions. But, as far as the official documents of both countries indicate, there is no cross-border cooperation in health care involving any hospitals or other facilities.

## Data and methods

### Study area

**Fig. 2:**
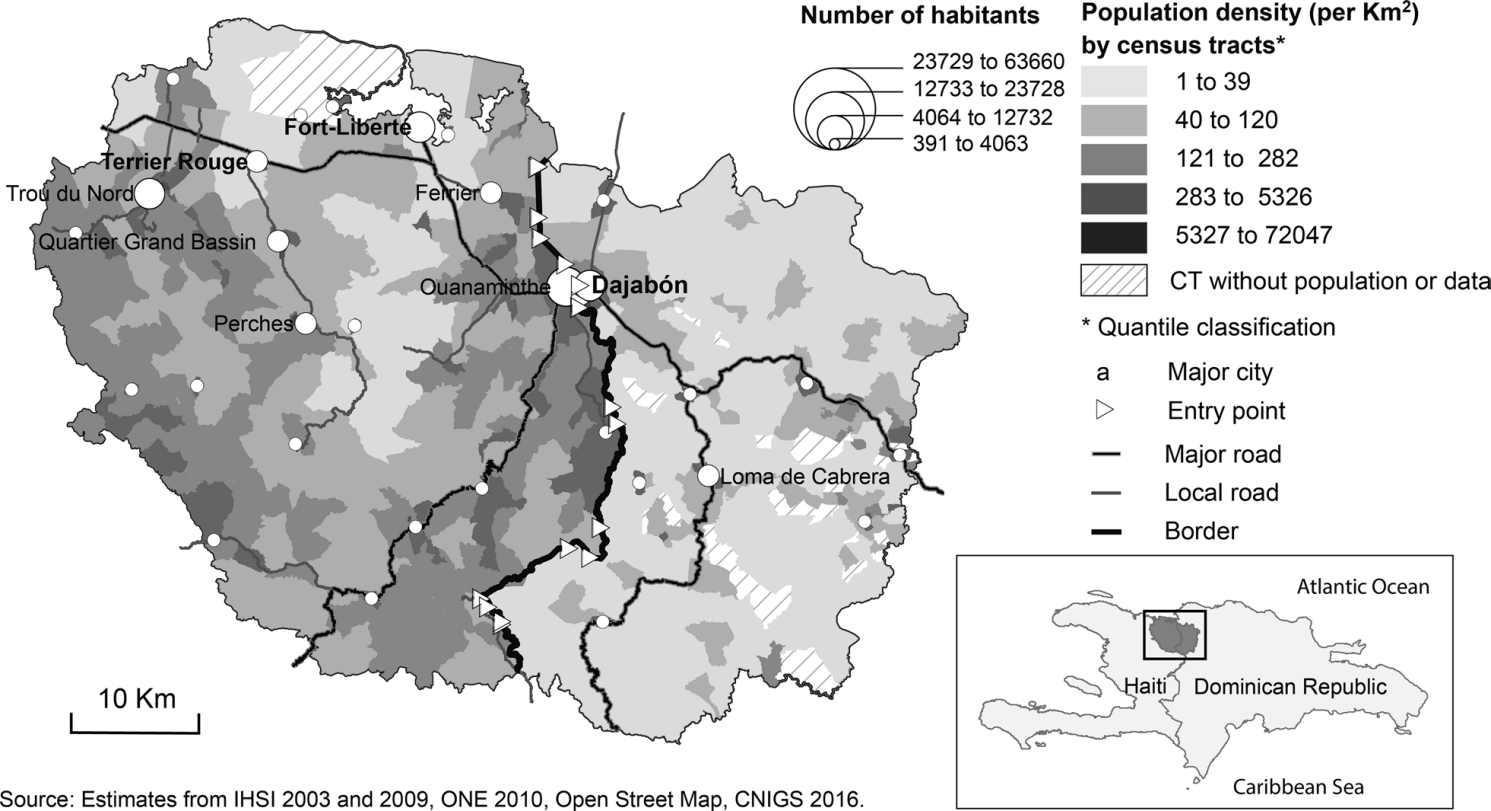
Study area

The area studied is in the northern part of the island of Haiti/Quisqueya, and focuses on the region along the Haitian-Dominican border, with one official daytime entry point (Ouanaminthe-Dajabón) and several informal crossing points (Fig. 2). Evidence for the last three decades has shown a significant growth in the intensity and diversity of interactions along the border, especially at the entry point, that is, Ouanaminthe-Dajabón [68, 69], the border’s second leading entry point.

The cities of Ouanaminthe and Dajabón have played an important role throughout the history of both countries [70]. They have also witnessed violent conflicts such as the massacre of thousands of Haitians in October 1937. This borderland is evolving nowadays from a barrier to a contact zone and an interdependent zone [41, 69, 70]. Several stakeholders (international organizations, the transnational capital, merchants, grassroots organizations, etc.) are engaged in this process [70]. The relocation of a private Dominican industrial free-trade zone in the fertile plain of Maribahoux in Ouanaminthe (a project financed by the International Finance Corporation) however highlights the advantages derived by the Dominican Republic from its different level of development from that of Haiti. It shows how such disparities are helping to widen the gaps and are fostering more asymmetrical interactions [41, 70, 71]. Furthermore, the recent proliferation of binational projects promoted and financed by international organizations is tending to set the framework for a new era of cross-border cooperation [41] in different fields, including health issues.

The level of poverty is globally higher in the borderland regions of both countries [72, 73], but there are still important disparities in terms of infrastructures, services, etc. between the North-East Department and the Province of Dajabón. As shown in Fig. 2, the Haitian side of the border is denser, with more and larger sized cities.

In the health sector, this intense mobility has forced the implementation of binational mechanisms for epidemiological surveillance. Gaps in the supply of social services (health, education) tend to lead to asymmetrical interactions and polarized flows in a push/pull dynamic [68, 69, 74]. On the other hand, few studies [49, 75] have indicated that the ratio of foreigners using health care facilities is higher in the Dominican borderland region compared with the rest of the country.

Statistics from the Dominican public health secretary show that, in 2015, almost 10% of public hospital patients (consultation and emergency) in the Province of Dajabón were foreigners. The rate is even higher for the primary care centres (35%) (Table 2). Information about foreign patients’ nationality is not available. The high percentage of Haitian migrants (87.2% of the immigrant population in Dominican Republic was born in Haiti according to the Second National Immigrant Survey held on 2017) and proximity to the border suggest that most of those foreign patients are Haitian, but there is no direct evidence for this. The condensed version of the Second National Immigrant Survey (ENI-2017) indicates that 77% of the migrants born in Haiti as well as 78% of those born in Dominican Republic of foreigners parents used the public health services [50], Moreover, hundreds of thousands of Haitian descendants [39] are not considered to be Dominicans because of the 2013 judgment TC/0168/13 of the Dominican Constitutional Court and the 169-14 Law [76]. It is thus difficult to estimate the percentage of patients crossing the border to obtain health care and the proportion of Haitians living in the Dominican Republic.

**Table 2:** Dominican health care facilities use for consultation and Emergency by national and foreign patients, 2015. *Source*: MSP, Vice Ministry of Planning and Development – Department of Health Information (DIS)

Health care facilities	National patients			Foreign patients		
	Consultation	Emergency	Total	Consultation	Emergency	Total
Hospital Municipal Partido*	6475	7337	13,812	610	380	990
Hospital Dr. Ramon Adriano Villalona*	15,478	4910	20,388	1265	420	1685
Hospital Municipal Restauración*	9881	2332	12,213	2902	472	3374
Hospital Ramon Matias Mella*	15,963	14,502	30,465	555	819	1374
First Level Centers (29) **	45,456	3068	48,524	24,568	1602	26,170

(29) equals to the number of first level centers/primary care units

*Database of monthly records of Hospitals Services (67A) 2015 updated on April 27th 2016

**Monthly reports of services of the centers of first level of attention (R-8) 2015

### Data

Three types of GIS data are needed to assess the potential accessibility of health care services.

**Fig. 3:**
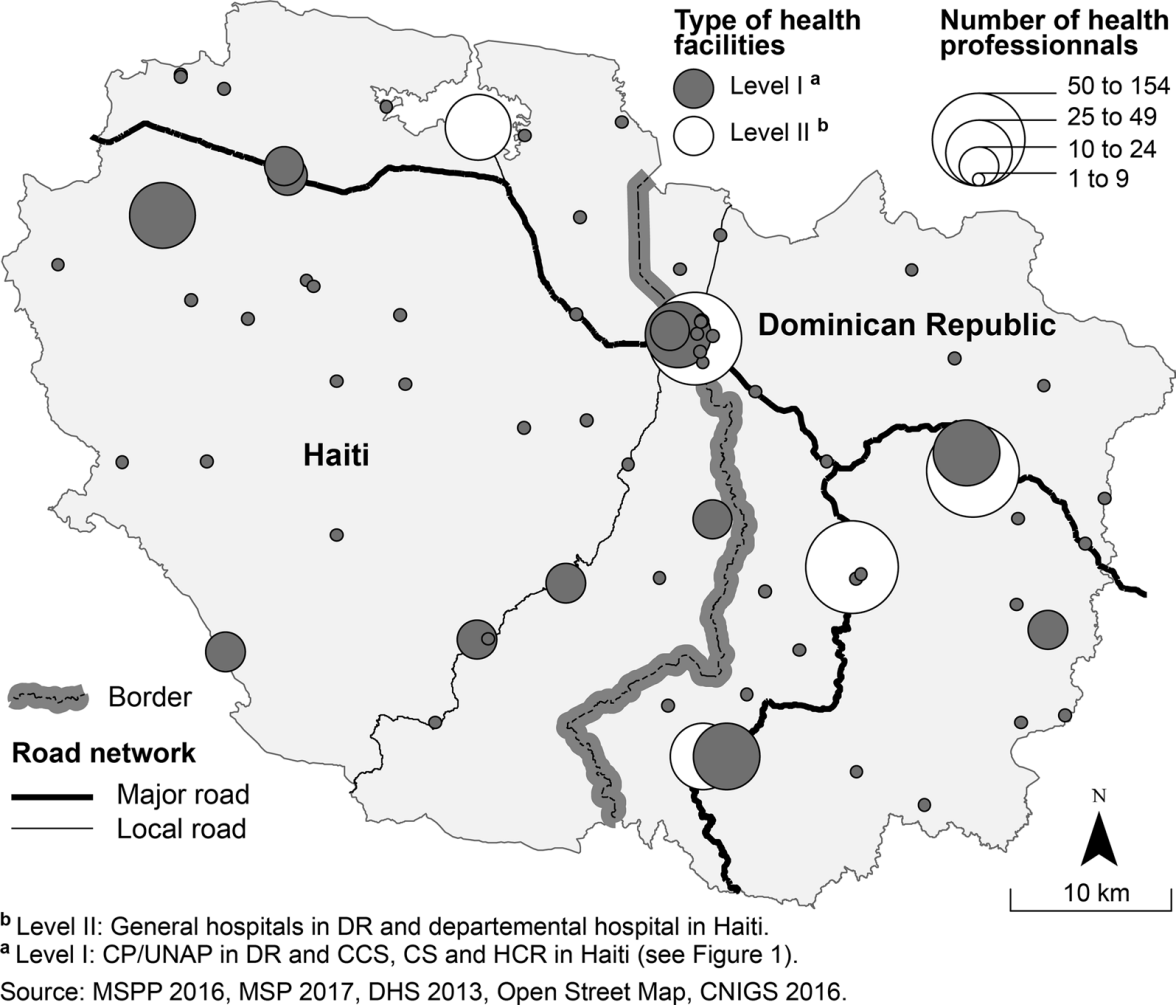
Health care facilities and health professionals in the studied area
- *For the demand side*: The demographic data have been extracted from the censuses at the equivalent of the census block level (*Section d’énumération*—*SDE*) for Haiti (N = 422) and at the neighbourhood level (*Barrio*) for the Dominican Republic (N = 202). The neighbourhood was the finest spatial unit available. The average population is 868 for the *SDE* and 317 for the *Barrio*. The demographic data for Haiti and the Dominican Republic were provided by the national statistical institutes (*Institut Haïtien de Statistiques et d’Informatique*—*IHSI* and *Oficina Nacional de Estadística*—*ONE*, respectively). Because the last census in Haiti was held in 2003, we had to estimate the population for 2010 (the year of the Dominican census). Our estimates are based on those made by the IHSI for 2009, in applying their population growth rate. The use of a centroid considers that the population is evenly distributed within the spatial unit used (*SDE* or *Barrio*), which is not the case, especially for a scattered rural population area. To better reflect the reality of the settlements in the rural areas, we use an adjusted centroid of the spatial unit. The adjustments are based on photo interpretations of Google and Bing imagery.
- *For the travel distance*: The road network data were retrieved from Open Street Map (OSM) for both countries. Data were also provided by Haiti’s National Centre of Geospatial Information (*Centre National d’Information Géospatiale* – *CNIGS*). The data were validated using Google and Bing imagery. The road classification of the Haitian and Dominican transport secretaries was used. A maximum travel speed was assigned to each class of road as indicated in Table 3 based on various sources and photointerpretation to assess roads conditions. For pathways, the maximum travel speed is 3 km/h in order to in some way reflect the geographic constraints, since the central part of the area studied is nested in a mountainous chain. The entry points were georeferenced based on aerial photo interpretation.

- *For the supply side*: The geographic locations of public health facilities in each country have been collected from the websites of the health secretaries of Haiti and the Dominican Republic. Data on the number of health professionals for each health facility have been provided by the Department of Information of the Dominican Republic’s public health ministry (*Departamento de Información de salud)*. For Haiti, such data were available on the health map on the website of the public health ministry (*Ministère de la santé publique et de la population)*. According to those respective sources, there is a total of 70 public health facilities (35 on each side) and 932 health professionals (322 on the Haitian side and 610 in the Dominican Republic) (Fig. 3). It is worth noticing on the Haitian side (North-East Department), there is only one public facility of the second level and none at the border city of Ouanaminthe. Meanwhile the Province of Dajabon counts with four public facilities of the second level (municipal hospitals or general hospital) and one of them located in the border city of Dajabón.

**Table 3:** Road classification and speed

Country	Road type	Speed
Haiti*	National roads	70 km/h
	Departmental roads and segment of national roads in living areas	50 km/h
	Communal roads, local roads, streets	30 km/h
	Track and others, unclassified roads	15 km/h
	Pathways	3 km/h
Dominican Republic**	Major roads and regional roads	80 km/h
	Local roads	50 km/h
	Streets	35 km/h
	Country roads	30 km/h
	Others, unclassified roads	15 km/h
	Pathways	3 km/h

*Sources*: *MTPTC 2015; CIAT 2010

**2010 at oisevi.org; DIGESETT, Ley 241-67

## Methods

To measure the impact of the opening of the border on the spatial access to health care, we consider different scenarios with varying border crossing time impedance: open, semi-open, or closed. These scenarios are hypothetical, since the level of control is not the same along the border or at the informal crossing points or entry points. Furthermore, different factors (objective and subjective) influence the smoothness of the flows of Haitians at the Dominican border.

The estimates of the time spent crossing are based upon: (1) on-site observations in July 2016 and June 2017 at the Ouanaminthe-Dajabón entry point; and (2) informative discussions with key resources in Ouanaminthe and organization members working along the border line.

- The first scenario is an open border, where there is less control (or almost none) at the Dominican border. The border is open on Friday and Monday when the so-called “binational” market takes place in Dajabón. Haitians are “free” to cross, and no papers are needed. But, due to the intense flows, delays could be observed. A 15-min cost is thus added to the travel time required to cross the border in order to take into account light traffic or migration controls.
- The second and third scenarios consider the border half closed. In this case, there is more control at the Dominican border. It is a twofold situation: a) a normal border control for migration and light traffic (scenario 2); and b) stricter control and heavy traffic (scenario 3). The cost varies from 30 min for scenario 2–60 min for scenario 3.
- In the fourth and last scenario, the border is closed. No crossing is permitted. This is, for example, the case during the night or in some other particular contexts such as conflicts, elections, etc.

For all four scenarios, we consider only one direction flow: from Haiti to the Dominican Republic. This choice is based on the hypothesis that, due to the disparities between both countries, a push/pull dynamic polarizes cross-border flows toward the Dominican Republic.

### The Enhanced Two-Step Floating Catchment Area (E2SFCA) method

The potential spatial accessibility as described earlier is the distance between the supply (in this case, the number of health professionals) and the demand, defined by the overall population. Numerous studies have demonstrated the importance of the distance (metres or travel time) to access health care in developing countries [1, 2, 4]. Geographic constraints as well as road conditions can trigger low access to health care and impact the use of health care facilities, with important repercussions for health outcomes and public health. Several methods are used to measure spatial accessibility [77, 78]. The approach based on available supply assumes that all users within the same catchment area have equal access regardless of the geographic constraints [9, 77]. The gravity model and its derived two-step floating catchment area (2SFCA) method consider spatial interactions and the mobility of the population [35]. The well-known two-step floating catchment area method computes the ratio between the supply (number of physicians or health professionals) and the demand (population) within a catchment area for each supply point at first and ultimately for each demand point [79, 80]. To overcome the limitations of the 2SFCA, an enhanced method has been developed by Luo and Qi [10] by applying weights to differentiate travel time zones in accounting for distance decay.

This method is used to evaluate the cross-border potential spatial accessibility of the health care services. Since the area studied includes rural areas, the catchment area (within a 60-min driving, motorbiking and walking time) has been divided into four travel time zones, as proposed by some authors [79, 81]: 0–15, 15–30, 30–45 and 45–90 min. The 45–90 min travel zone considers the 60-min cost for a semi-open border with stricter control, as indicated above. The maximum travel speed for each class of road accounts for the assumed mixed transportation mode (walking combined with motorbiking, the most usual transportation mode in the studied area).

The method is implemented in two steps, using the equations below. The first step assigns an initial ratio to each health service within the catchment area. In the second step, for each demand location within the catchment area, we search all supply locations and then sum up the initial ratio *R*_*j*_ at these locations. The resulting *A*_*k*_ represents the accessibility of the population at location *k*, *R*_*j*_ the supply-to-population ratio at the health service (supply) location *j* that falls within the catchment area, and *d*_*kj*_ the distance (min) between *k* and *j*. The same distance weights derived from the Gaussian function used in step 1 are applied to different travel time zones to account for distance decay. A larger value implies better accessibility.

$$R_{j} = \frac{{S_{j} }}{{\sum\limits_{{k \in \left\{ {d_{kj} \in D_{r} } \right\}}} {P_{k} W_{kj} } }} = \frac{{S_{j} }}{{\sum\limits_{{k \in \left\{ {d_{kj} \in d_{1} } \right\}}} {P_{k} W_{1} } + \sum\limits_{{k \in \left\{ {d_{kj} \in d_{2} } \right\}}} {P_{k} W_{2} } + \sum\limits_{{k \in \left\{ {d_{kj} \in d_{3} } \right\}}} {P_{k} W_{3} } + \sum\limits_{{k \in \left\{ {d_{kj} \in d_{4} } \right\}}} {P_{k} W_{4} } }}$$

$$A_{k} = \sum\limits_{{k \in \left\{ {d_{kj} \in D_{r} } \right\}}} {R_{j} } = \sum\limits_{{k \in \left\{ {d_{kj} \in d_{1} } \right\}}} {R_{j} W_{1} } + \sum\limits_{{k \in \left\{ {d_{kj} \in d_{2} } \right\}}} {R_{j} W_{2} } + \sum\limits_{{k \in \left\{ {d_{kj} \in d3} \right\}}} {R_{j} W_{3} } + \sum\limits_{{k \in \left\{ {d_{kj} \in d_{4} } \right\}}} {R_{j} W_{4} }$$

where *S*_*j*_ represents the weight given to service *S* such as its size (i.e. number of health professionals) (“supply side”), *d*_*kj*_ is the distance (travel time) between spatial unit centroid *k* and health service *j*, *d*_0_ is the threshold travel time (min), *P*_*k*_ represents the demand at location *k* that falls within catchment area *j* and *W*_1_, *W*_2_, *W*_3_, *W*_4_ = 1.00, 0.80, 0.55, 0.15 with a slow step-decay function or 1.00, 0.60, 0.25, 0.05 with a fast step-decay function.

The calculations are done using two kinds of software (ArcGIS and SAS). The cost-distance matrix obtained using the Network Analyst extension in ArcGIS has been exported to SAS to compute the E2SFCA. The final results are mapped in ArcGIS.

Statistical analysis was conducted to explore the differences and variation for the E2SFCA calculations. The Wilcoxon test was computed to assess the differences and variation observed in the E2SFCA results for each scenario and country. Finally, linear regression models were used to predict the accessibility of health services (E2SFCA) according to the four scenarios and their variation. All statistical analyses were carried out using SAS software.

## Results

**Fig. 4:**
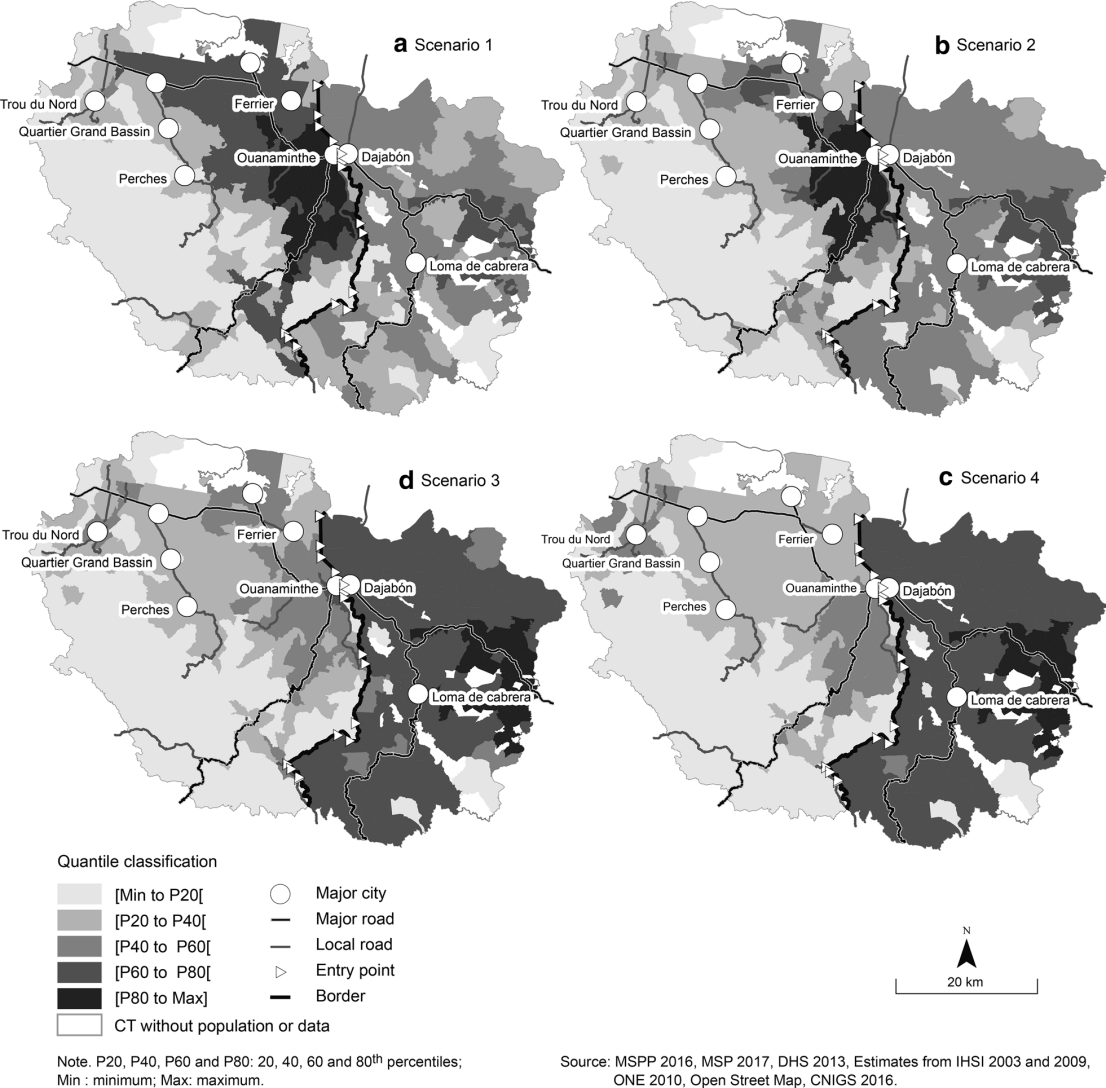
E2SFCA results

As mentioned before, four simulations are considered to measure the impact of the opening of the border on the level of accessibility of public health services for the borderland population of the North-East Department and the Province of Dajabón. To facilitate the comparison between the four scenarios, a quantile classification with five classes has been used and mapped (Fig. 4). Following are the results for each scenario.

### Scenario 1: Open border

The first scenario is with an open border. A penalty of 15 min is added to the travel time of Haitians crossing the border. The results show contrasting levels of accessibility in the North-East Department between areas next to the border and more remote locations (Fig. 4a). Two features stand out. First, a large area located mostly in the commune of Ouanaminthe has the highest ratio of accessibility. A smooth gradation is observed to the west (along the national road connecting this region with the North Department and its capital, Cap-Haïtien, the second most important city in Haiti), and to the northwest toward Fort-Liberté (the North-East Department’s capital). Second, a sharp drop in the level of accessibility is seen between those two regions (respectively [P60 to P80[and [P80 to Max], the last two quintiles) and the other remote locations (corresponding to the first quintile, [Min to P20[). The areas with the highest level of accessibility are those where hospitals with a larger number of health professionals are located. They are also better connected to a road network in good condition, with higher maximum speeds.

The pattern in the Dominican Republic is quite different: the municipalities at the edge of the Province have the highest level of accessibility, and those next to the border have moderate to low access. There are scattered areas with a very low level of access to health care. Dajabón, the main city of the Province, has a moderate level of accessibility with an open border because of its proximity to Ouanaminthe, a city with a population of 60,000. Therefore, an open border induces potential overload of the Dominican health care services due to an increased demand from Haitians and consequently lowers the health professional-to-population accessibility ratio for the Dominicans. But the overall situation in terms of accessibility in the Dominican Republic remains better than in Haiti, even with an open border.

### Scenario 2 and scenario 3: Half-closed border

Scenario 2 is with a half-closed border, with a 30-min cost to cross the border, and the scenario 3 is with a 60-min cost. The map indicates some changes in the pattern compared with the open border (Fig. 4b). First, there is a small drop in the extent of the area with the highest accessibility on the Haitian border side. Second, on the Dominican side, the level of accessibility is globally higher than that observed in the first scenario because of a decrease in the potential demand from Haitians at the Dominican sites.

Scenario 3 is a half-closed border, with a 60-min cost added for crossing the border, indicating more control on the Dominican border (Fig. 4c). The results show a significant reduction in the extent of the area with a higher level of accessibility on the Haitian side of the border. On the Dominican side of the border, there is a noticeable improvement in the overall level of accessibility in the Province of Dajabón. The 60-min cost added causes a significant decrease in the Haitians’ potential demand at the Dominican sites which is limited to the 15 min travel zone. Therefore, the Dominicans accessibility level increases beyond the 30–45 min travel zones. The results also emphasize the impact of the low road coverage especially on the Haitian population’s access to health resources.

### Scenario 4: Closed border

With the border closed, the results show the potential spatial accessibility of health care facilities in each country (Fig. 4d). Globally, the level of accessibility is higher in the Dominican Republic than in Haiti. In fact, the quasi-totality of the Haitian spatial units belongs to the first two quintiles (light gray), while those of the Dominican Republic belong to the last two quintiles (dark gray), drawing attention to the existing disparities between both countries in terms of potential accessibility to health care. This scenario also confirms the striking gaps within the North-East Department, especially between the remote locations and the urban areas.

### Variation between scenario 4 and scenario 1

**Fig. 5:**
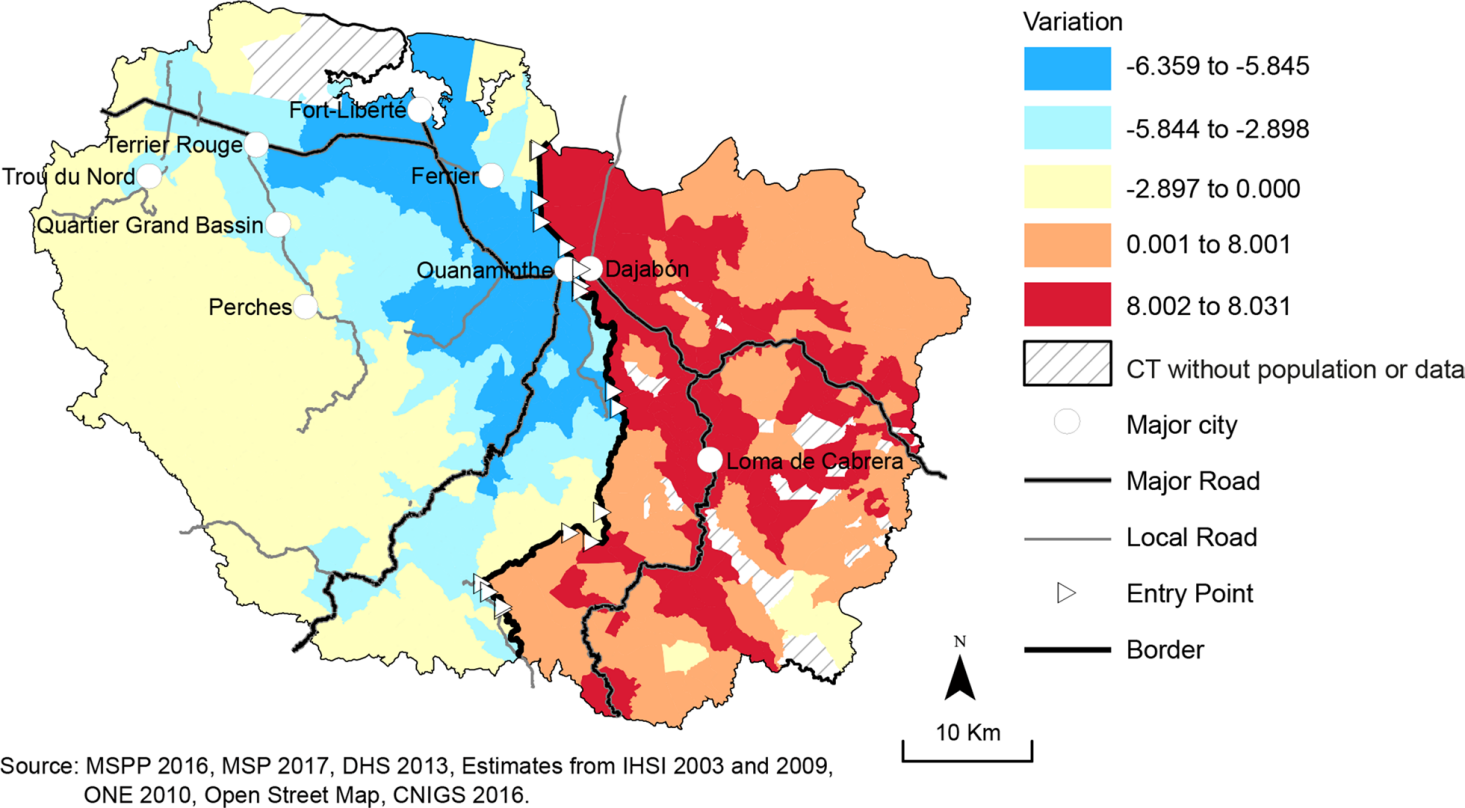
Variations in E2SFCA Results for Scenario 4 versus Scenario 1

Figure 5 shows the variation in the level of spatial accessibility between scenario 4 (closed border) and scenario 1 (open border). It highlights the areas most affected by the border’s level of openness. As shown in Fig. 5, the solid blue areas are those that benefit from an open border. The red ones are those gaining better access when the border is closed. The border has almost no impact on an extended territory (pale yellow) of the North-East Department where the variation differences are negative but close to zero.

In both countries, the areas next to the border are those that are more sensitive to the impact of the border on their level of spatial accessibility. Those areas are the ones where an open border induces an increased demand from Haitians at the Dominican health services located near the border (within the 15–45 min travel zone). It is also important to note the importance of the road network in the border effect, as the pattern is aligned with the main road network. For example, borderland areas (southern part of the North-East Department in Haiti) covered with pathways and with geographic constraints don’t benefit at the same level as those with a good road network coverage. A similar sensibility pattern is observed in the Province of Dajabón.

### Results of nonparametric test and regression models

**Fig. 6:**
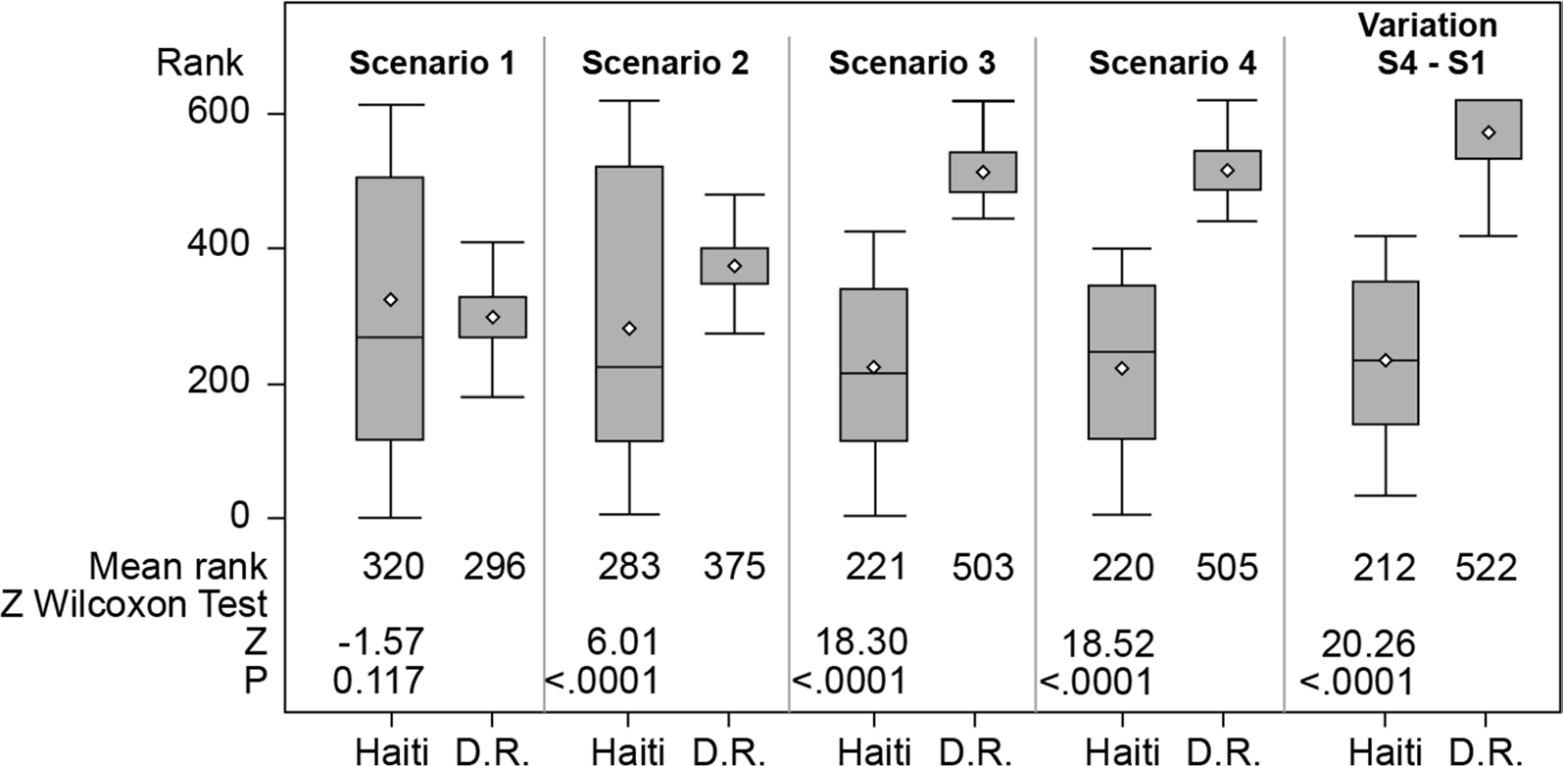
Boxplots for the four scenarios

To explore differences (location and scale) and variation in the E2SFCA results for each country, we conduct a nonparametric test (Wilcoxon test). Figure 6 shows that, scenarios 2 (mean rank = 283 for Haiti vs 375 for Dominican Republic, *z* = 6.01, *p *< 0.0001) to 4 (mean rank = 220 for Haiti, 505 vs Dominican Republic, *z* = 1.52, *p *< 0.0001), as well as for the variation (scenario 4–scenario 1) (mean rank = 212 for Haiti vs 522 for Dominican Republic, *z* = 20.26, *p *< 0.0001), the results are significant (*p *< 0.0001), but that is not the case for scenario 1 (mean rank = 320 for Haiti vs 296 for Dominican Republic, *z* = − 0.17, *p* = 0.117). It is relevant to note: a) the dispersion of the scores for Haiti compared to those for the Dominican Republic; and b) the gap in mean rank between Haiti and the Dominican Republic for scenario 3 (border half-closed) and scenario 4 (closed border). The variability and dispersion in the range for Haiti emphasize the disparities within the North-East Department shown in Fig. 4. The results for the variation between an open border and a closed border confirm the impact of the border on the level of spatial accessibility of health care for the Haitian population.

Finally, several linear regression models are conducted to predict the accessibility of health services (E2SFCA results) according to the four scenarios and variation between the two extremes. Two independent variables are introduced in these models: Haiti (D.R. is defined as the reference category), and rural area (versus urban area). The results of these models are shown in Table 4.

**Table 4:** Linear regression for E2SFCA (n = 624)

Scenarios	Coefficient			
	Intercept	Haiti^a^	Rural^b^	R^2^
Scenario 1	7.98	− 0.68 **	− 2.33 ***	0.15
Scenario 2	10.54	− 3.03 ***	− 2.82 ***	0.24
Scenario 3	13.60	− 8.47 ***	− 2.37 ***	0.68
Scenario 4	14.10	− 11.49 ***	− 0.79 ***	0.89
Δ Scenario 4–Scenario 1	6.12	− 10.81 ***	1.54 ***	0.85

Signif. codes:: *** 0.001, ** 0.01

^a^Reference: Dominican Republic

^b^Reference: urban

First, note that R^2^ increases from 0.15 to 0.89 for scenarios 1–4. Next, the degree of border openness has a significant impact on accessibility on both sides of the border, to the detriment of Haiti (with increasingly strong negative regression coefficients). Not surprisingly, the coefficients for rural areas confirm that these areas have poorer accessibility, regardless of the scenario. In addition, the positive and significant coefficient for the variation between scenarios 4 and 3 shows that the closure of the border strongly affects accessibility in urban centres that are close to the border.

## Discussion

The E2SFCA results and statistical analyses clearly highlight the impact of the border on the potential spatial accessibility of public health services for Haitian and Dominican border populations with a peculiar pattern caused by the one directional movement assumed for the model. In fact, the simulations carried out show that Haitian populations in areas close to the border line—particularly near an entry point (formal or informal)—and served by a road network in good condition have higher levels of accessibility when the border is open (scenario 1) or semi-closed (scenario 2), with a 30-min penalty. At the same time, an increased demand from Haitians of those specific areas for the Dominican health services lowers the health professional to population accessibility ratio in Dominican Republic causing striking variations according to the openness of the border. It is therefore interesting to note that, by increasing the cost from 30 to 60 min, the level of accessibility varies widely across the border. Thus, the opening of the border only impacts spatial accessibility for the Haitian population in the vicinity (travel time zones 0–15 min and 15–30 min). These results are not surprising, as these areas have a road network in good condition, confirming the importance of a good road network [4, 82–84] and of the type of distance [78] in potential spatial accessibility. As a result, rural areas are those with the lowest level of accessibility, on the one hand, and, on the other hand, these areas benefit very little from the opening of the border, despite its proximity. A weak road network (absence of roads or roads in poor condition) and topographical constraints associated with a limited offer of services (type of service and number of health professionals) indeed characterize Haitian rural areas. In Dominican Republic, an open border besides creating as mentioned before a decrease in the level of accessibility generates more disparities within the Province of Dajabón, especially for the population at its edges. Introducing a 30 or a 60-min cost for a semi-closed border smoothens the gaps within Dajabón since the Haitians’ demand at the Dominican health services decrease.

Scenario 4 highlights the differences in the potential spatial accessibility of health services between the two countries. These differences clearly underline the health and spatial discontinuities due to the border. The disparities in the spatial accessibility of public health services are very low (or almost non-existent) within the Dominican territory, in striking contrast with Haiti, where they are high. Those gaps can lead to a one-directional flow like the one assumed by the model. Furthermore, several empirical studies [16, 85, 86] in different border contexts indicate a pattern of polarized flows because of an unsatisfied demand in one side and a more attracted one on the other side. Nevertheless, this push/pull dynamic could have considerable impact on the health services of the recipient country depending on their public health care capacity, the volume of cross-border patients and the borderland context including the level of cooperation or integration of the countries involved. It is worth noticing that the challenges for both countries regarding those issues are high even more when considering the results of the potential spatial accessibility model.

However, an optimization of the E2SFCA to weight the population according to the real use of health services on both sides of the border would have given a closer insight into the reality of potential spatial accessibility. It would also have been appropriate to assess the impact of the border on potential spatial accessibility by integrating socioeconomic and demographic factors to analyze the correlation between population characteristics and cross-border spatial accessibility.

The results also call for better cooperation and integration of the two countries’ health care systems. In this regard, the stakes for Haiti and the Dominican Republic are high, not only because of the instability of relations between the two countries, but also because of the thorny issue of migration. As Alexandre [48] points out, cross-border movements between Haiti and the Dominican Republic, including movements linked to health, cannot be thought of without considering a reform of the migration legislation in both countries.

## Conclusion

The results emphasize the impact of a good road network on the spatial accessibility of health care, as discussed in many studies. They also show the impact of the openness of an international border on the potential accessibility of health care in borderland regions, highlighting the importance of distance. Proximity is thus seen as one of the determinants in cross-border mobility and in health care seeking behavior. But other factors such as the attractiveness (quality, cost) of health care services must be considered to analyze individuals’ behaviors. In our research, we also assume that all the Haitian population of the North-East Department would potentially choose to cross the border, but this is not actually the case. An optimization of the model would make it possible to better evaluate the impact of the border and to obtain more robust results, with a better appreciation of the reality of the situation. A gender-oriented analysis could also have been of interest considering, inter alia, the high maternal mortality rate in Haiti and the high number of unassisted deliveries, particularly in the rural Haitian areas.

The study also highlights the need for more research so as to better understand the determinants of cross-border health care use. Moreover, the distance thresholds are arbitrary and do not necessarily reflect specific patients’ behavior, suggesting the need for qualitative inquiry to assess the therapeutic. In-depth interviews and surveys could therefore offer an insight into revealed spatial access and lead to a better understanding of patients’ behavior and how this is related to their practices around the border.

Furthermore, cross-border movements in health are part of bigger issues. They should be addressed not only in shrinking the gaps in health access resources but also in creating the needed legal and institutional environment for them to develop smoothly.
